# Factors Associated with Self-Medication during the COVID-19 Pandemic: A Cross-Sectional Study in Pakistan

**DOI:** 10.3390/tropicalmed7110330

**Published:** 2022-10-25

**Authors:** Bakhtawar Chaudhry, Saiza Azhar, Shazia Jamshed, Jahanzaib Ahmed, Laiq-ur-Rehman Khan, Zahid Saeed, Melinda Madléna, Márió Gajdács, Abdur Rasheed

**Affiliations:** 1 College of Pharmacy, University of Sargodha, Sargodha 40100, Pakistan; 2 Department of Clinical Pharmacy and Practice, Faculty of Pharmacy, Universiti Sultan Zainal Abidin, Kuala Terengganu 21300, Malaysia; 3 Department of Oral Biology and Experimental Dental Research, Faculty of Dentistry, University of Szeged, 6720 Szeged, Hungary; 4 School of Public Health Dow, University of Health Sciences, Karachi 74200, Pakistan

**Keywords:** self-medication, COVID-19, pandemic, over-the-counter, medicine use, Pakistan

## Abstract

Self-medication (SM) is characterized by the procurement and use of medicines by bypassing primary healthcare services and without consulting a physician, usually to manage acute symptoms of self-diagnosed illnesses. Due to the limited availability of primary healthcare services and the anxiety associated with the COVID-19 pandemic, the compulsion to SM by the public has increased considerably. The study aimed to assess the characteristics, practices, and associated factors of SM by the public during the COVID-19 pandemic in Sargodha, Pakistan. χ^2^-tests and univariable analyses were conducted to explore the identification of characteristics and the potential contributing factors for SM during COVID-19, while multivariable logistic regression models were run to study the effect of variables that maintained a significant association. The study was performed during July–September 2021, with *n* = 460 questionnaires returned overall (response rate: 99.5%). The majority of respondents were males (58.7%, *n* = 270) who live in the periphery of the town (63.9%, *n* = 294), and most of the respondents belonged to the age group of 18–28 years (73.3%, *n* = 339). A large number, 46.1% (*n* = 212), of the participants were tested for COVID-19 during the pandemic, and among them, 34.3% (*n* = 158) practiced SM during the pandemic; the most common source of obtaining medicines was requesting them directly from a pharmacy (25.0%; *n* = 127). The chances of practicing SM for medical health professionals were 1.482 (*p*-value = 0.046) times greater than for non-medical health personnel. The likelihood of practicing SM in participants whose COVID-19 test was positive was 7.688 (*p*-value < 0.001) times more than who did not test for COVID-19. Allopathic medicines, acetaminophen (23.6%), azithromycin (14,9%), and cough syrups (13%), and over the counter (OTC) pharmaceuticals, vitamin oral supplements, such as Vitamin C (39.1%), folic acid (23.5%), and calcium (22.6%), were the most commonly consumed medicines and supplements, respectively; being a healthcare professional or having a COVID-test prior showed a significant association with the usage of Vitamin C (*p* < 0.05 in all cases). Respondents who mentioned unavailability of the physician and difficulty in travelling/reaching healthcare professionals were found 2.062-times (*p*-value = 0.004) and 1.862-times (*p*-value = 0.021) more likely to practice SM, respectively; SM due to fear of COVID was more common in individuals who had received COVID-tests prior (*p* = 0.004). Practices of SM were observed at alarming levels among our participants. Consciousness and understanding about the possible adverse effects of SM must be established and validated on a continuous level; in addition, on a commercial level, collaboration from pharmacists not to sell products (especially prescription-only medicines) without a certified prescription must be developed and implemented.

## 1. Introduction

Self-medication (SM) is characterized by the procurement and use of medicines by bypassing primary healthcare services and without consulting a physician, usually to manage acute symptoms of self-diagnosed illnesses [1,2]. Based on the World Health Organization’s (WHO) definition, SM is “the choice and use of drugs by any individual in order to treat their own self-identified illness or symptoms” [3]. Drugs used for SM normally include over the counter drugs (OTC), however, in some cases (when the patients acquire them from various sources) prescription-only medicines (POM) are also relevant [4]. The intention of utilizing SM may be affected by various factors, such as individual, organizational, and environmental variables [5]. Individual factors include age, income, gender, highest level of education, life satisfaction, convenience, and urgency/severity of symptoms [6]. Commercials and adverts by pharmaceutical companies via the media and the internet also have a considerable role in facilitating this practice [7]. SM incorporates purchasing drugs (both from formal and informal sources), or re-utilizing stashes (i.e., leftovers from a medicine cabinet) from past prescriptions, receiving medicines from and taking them on the counsel of relatives, neighbors, and friends [8]. SM is a global public health issue; nevertheless, the prevalence of this practice is more common in developing countries (i.e., low and middle-income countries) [9,10]. In these regions, organizational attributes, such as poor quality and availability of healthcare services, a relatively high number of individuals without health insurance, a lack of human resources, unavailability of transport services, non-professional behaviors of healthcare providers, and long turnaround times—coupled with the availability of drugs for purchase from “hawkers”—considerably increase the SM [11,12]. The lack of knowledge regarding the use of pharmaceuticals (i.e., their indications, dosage, appropriate treatment duration, and possible side effects) and mistrust towards physicians may also facilitate SM [13,14]. Although the WHO has noted that the practice of SM may remedy some minor obsessive situations at a reasonable expense, there have been reports that it might lead to the squandering of medical assets and excess pharmaceutical waste [15]. In addition, inappropriate use of pharmaceuticals carries the risk of a delayed diagnosis, an unfavorable response to medications, excess morbidity, and the emergence of multi-drug resistant (MDR) organisms in the case of antimicrobials [15,16,17]. The general population of Pakistan turned to self-medication and symptomatic therapy because of inadequate care for the COVID-19 infection; about 80% of the population also stockpiled drugs for use during the pandemic [18].

During the first part of 2020, the WHO cautioned the world about the rapid spread of the novel coronavirus (SARS-CoV-2), which later progressed into a global pandemic; due to the associated disease (COVID-19), an overall lockdown was set off in the greater part of the world [19]. The pandemic has caused a considerable burden on healthcare infrastructures worldwide, especially in countries where the healthcare framework was fragile to begin with [20]. In response to the limited availability of primary healthcare services and the anxiety associated with the pandemic, the compulsion to SM by the public has increased considerably, as in the eyes of many, this was the only sensible “link” to healthcare [21,22]. In parallel with the onset of the pandemic, many studies (both pre-clinical and clinical) have been published on the effectiveness of various drugs in the treatment and prevention of COVID-19; these included anti-malarial agents (chloroquine and hydroxychloroquine), antibiotics (azithromycin and doxycycline), antiparasitic drugs (ivermectin), decongestants (azelastine), leukotriene inhibitors (montelukast), non-steroidal anti-inflammatory drugs, and acetaminophen, alongside nutrients, such as Vitamin C and D, zinc, and calcium [23]. Although the effectiveness of most of the above mentioned therapies has largely been disproven by multicentric clinical trials, in the first and second waves of the pandemic—in combination with the rampant “infodemic” regarding COVID treatments in online media—attempts to treat COVID-19 with e.g., hydroxychloroquine in the absence of any healthcare professional consultation or prescription (as a prime example of SM) were widespread [24,25,26]. Due to the overlap of symptoms between COVID-19 and other viral respiratory infections (e.g., throat aches, dry cough, malaise, fever, and shortness of breath), in many regions, individuals began taking drugs without being tested for COVID-19 at all, often leading to drug shortages due to supply chain issues [27].

Since its global spread in 2020, COVID-19 has led to considerable morbidity and mortality, significant upheaval in healthcare systems worldwide, and the fear of infection has been constantly present in the lives of individuals; this has led to anxiety and tension in both medical service laborers and the overall population in numerous parts of the world [28]. These factors may have contributed to an increase in SM; thus, the present study aimed to investigate the characteristics, practices, and potential contributing factors towards the use of SM during COVID-19 in Sargodha, Pakistan. This research also explored the different types of medicines used for SM during COVID-19.

## 2. Materials and Methods

### 2.1. Study Design, Study Site and Population

A questionnaire-based cross-sectional study design was adopted to assess the characteristics, practices, and contributing factors towards SM during the COVID-19 pandemic in Sargodha, Pakistan (155 km^2^, 12th largest city by population, with ~660,000 inhabitants and a literacy rate ~80%). The potential population of this study was the general population of Sargodha city and its periphery. The respondents or participants were selected through convenience and snowball sampling methods. The study was conducted between July and September 2021.

### 2.2. Sample Size Calculation

To establish the required sample size for our study, a sample size calculation was performed by using the Raosoft sample size calculator [29,30], based on the Formula (1) below:

(1) $$n=N\frac{x}{\left(N-1\right)E2+x}$$

where the population *N* was set at 20,000 (as the general population of Sargodha city was >20,000; however, in such population ranges, higher population values do not have an effect on the target sample size), *x* is the confidence interval of 95%, *E* is the margin of error set at 5%, and the expected response rate is set at 50%. 

The calculated initial sample size of residents of Sargodha was 384, which was increased by 20% for added contingency (to adjust for factors such as withdrawals, missing and incomplete questionnaires), with the final sample size set at *n* = 462.

### 2.3. Study Instrument and Data Collection

Before the development of the research instrument, a literature search was performed to ascertain potentially relevant questions and topics; during this process, we converted the research topic into keywords, which served as the foundation of an efficient search by providing results based on any of the terms included. After a thorough search of the literature, a structured and validated questionnaire was developed as a data collection tool. The questionnaire was validated by experts and researchers and for a better understanding of the respondents, then an interviewer-administered technique was used. The questionnaire was comprised of statements and items pertaining to the following sections: (i) socio-demographic data and general questions about the participants, including whether they are healthcare professionals or their history of being COVID tested; (ii) knowledge, attitudes, and practices towards SM during the COVID-19 pandemic, types of medicines used for SM; and (iii) potential contributing factors influencing SM. The translation and adaptation of the questionnaire were performed according to the criteria of Beaton et al. [31]. Before the main study, pilot testing was performed (involving 30 participants not included in the sample population) for the instrument to assess its face and content validity and comprehension/readability by the respondents. Using Cronbach’s Alpha, the instrument’s internal consistency and reliability were evaluated; the resultant value (α = 0.710) showed acceptable reliability in questionnaire-based research. Based on the experiences from the pilot testing of the questionnaire, various minor changes have been made in the wording of the paper questionnaire to produce the final instrument (Supplementary Material S1). The final instrument was then administered by the interviewer, which meant that the principal researcher approached each participant personally, and the interviewer gave the respondent feedback or repeated the question or available options (if an invalid one was given) to obtain an appropriate response. Each participant was explained the nature of the study and asked their responses. If any query arose at that time, the principal researcher clarified the doubts and proceeded with data collection. 

### 2.4. Inclusion and Exclusion Criteria

The participants included in this study were willing adults (between 18 and 60 years of age) without having any communication problems, either due to illness or some other reason. Adults who could not participate without a caretaker or guardian, and people approached who were unwilling to participate were excluded. 

### 2.5. Statistical Analysis

Data analysis—including descriptive statistics (frequencies, means, and percentages) and all inferential statistical analyses were performed using SPSS (Statistical Package for Social Sciences) version 24 (SPSS Inc., Chicago, IL, USA). χ^2^-tests and univariable analyses were conducted to explore the identification of characteristics and the potential contributing factors for self-medication during COVID-19. Multivariable logistic regression models were run to study the effects of variables that maintained a significant association. Results are presented as odds ratios (OR) and 95% confidence intervals (CI); *p*-values ≤ 0.05 were considered statistically significant. 

### 2.6. Ethical Considerations

The study was conducted in accordance with the Declaration of Helsinki and national and institutional ethical standards. Study approval for the study protocol was obtained from the Advanced Studies and Research Board of the University of Sargodha (Ref number: SU/Acad/1723). All participants were informed of the nature and aims of the study and the data collected; all willing participants of the study signed an informed consent form. The confidentiality and anonymity of the participants were protected throughout the study.

## 3. Results

### 3.1. Socio-demographic Characteristics of the Participants

Out of the 462 questionnaires, *n* = 460 questionnaires were returned completely filled out, resulting in a response rate of 99.5%. The socio-demographic characteristics of the study participants are summarized in Table 1; participants were invited to add their age in years, but later it was binned to groups. The majority of respondents were males (58.7%, *n* = 270) who lived in the periphery of the town (63.9%, *n* = 245), and most of the respondents belonged to the age group of 18–28 years (73.3%, *n* = 339). Only 46.1% (*n* = 212) of the participants were tested for COVID-19 during the pandemic. Almost half (46.5%, *n* = 214) of the respondents were working in the healthcare field. 

### 3.2. Characteristics of SM during the COVID-19 Pandemic

Table 2 presents our main findings regarding the practices of SM in our study population. Overall, 34.3% (*n* = 158) of participants self-medicated during the COVID-19 pandemic. The most common sources of drugs for SM were from requesting them directly from a pharmacy (25.0%). A significant association was observed between responses to SM, being employed in the healthcare profession (*p* = 0.046), and being tested for COVID-19 (*p* < 0.001). The types of medicines (allopathic vs. others and OTC and POM vs. POM only) used for SM were associated with area of residence and COVID-testing (*p* < 0.001). The majority of the respondents, about 65.9% (*n* = 304), were aware of the possible adverse effects of the SM drug taken, there was a significant association found with being employed in the healthcare profession (*p* < 0.001) and being tested for COVID-19 (*p* = 0.004).

### 3.3. Types of Medicines Used as SM during the COVID-19 Pandemic

The types of medicines used as SM and the associated correlates with their use were summarized in Table 3. The use of herbal medicines as SM was prevalent among respondents: 51.7% of participants never used any herbal medicines, while 20.0 % used Senna Makhi Kehwa; their use was significantly associated with area of residence (*p* < 0.001), being affiliated with the medical profession (*p* < 0.001) and undergoing a test for COVID-19 (*p* < 0.017). Among allopathic medicines, the most commonly used drugs were acetaminophen (23.6 %), azithromycin (14,9 %), and cough syrups (13.0 %), all drugs associated with the SM for the prevention and treatment of COVID infections during lockdowns; area of residence, being a healthcare professional, or having a COVID-test were important correlates. The most commonly consumed supplements during COVID-19 in our sample were vitamins (Vitamin C: 39.1%, folic acid: 23.5%, and calcium: 22.6%); area of residence, being a healthcare professional, or having a COVID-test showed significant correlation (*p* < 0.001) with the use of these supplements to boost immunity against COVID.

### 3.4. Possible Contributing Factors and Reasons Associated with SM during the COVID-19 Pandemic

Potential contributing factors for SM were identified based on the literature review of factors shown to increase SM, and an expert consensus of a group of public health specialists. Reported reasons and contributing factors associated with SM in our sample are shown in Table 4. While previously existing SM habits (7.3%) were also noted, the main reasons for SM were identified, i.e., the unavailability (13.9%) and difficulty in travelling/reaching healthcare professionals (12%), which may have led to preventive SM. SM associated with unavailability of a physician was more common in the peripheral parts of the city (*p* = 0.010), while SM due to difficulty in travelling/reaching healthcare professionals was more common in individuals who had received COVID-tests prior (*p* = 0.030). 

Table 5 depicts the results of univariable and multivariable logistic regression analyses. Univariable analyses showed that the likelihood of practicing SM for individuals within the age group of 29–38 years was 1.72-fold (*p*-value = 0.047) compared to participants between 18–28 years of age. The chance of practicing SM for medical health professionals was 1.482-times higher (*p*-value = 0.046) than for non-medical health professionals. The likelihood of practicing SM for participants whose COVID-19 test was positive was 7.688-times (*p*-value < 0.001) more than for those who did not test for COVID-19. Individuals who were aware of the possible side effects of SM drugs were 2.266-times (*p*-value < 0.001) more likely to practice SM. Participants who received information regarding the possible side effects of the SM by the physician showed an almost 2 times (*p*-value = 0.045) higher chance of performing SM practices as compared to those who did not receive such information. As far as reasons are concerned, individuals who mentioned unavailability of the physician and difficulty in travelling/reaching healthcare professionals were found 2.062-times (*p*-value = 0.004) and 1.862-times (*p*-value =0.021) time more likely to perform SM, respectively. 

The selection of variables for multivariable logistic regression analyses was based on the significance of the variables (*p*-values ≤ 0.05) in the univariable analysis. Furthermore, to confirm the best fitted model, different multivariable logistic models were run, for example, a multivariable model included all variables that were presented in univariable analysis, and another multivariable model included only those variables that were significant in univariable analysis. With the help of Akaike Information Criterion, AIC (the minimum values are better), it was found that the multivariable model presented in Table 5 was found to be better; the adjusted odds ratio and confidence interval are also reported in Table 5. Hence, after adjusting variables no. 1, 2, 3, 4, 5, 6, 8, 10, and 14 listed in Table 5, it was noted that variables 3 (“Tested for COVID”) and 4 (“Were you aware of the possible side effects of the SM drugs?”) showed significant (*p*-values ≤ 0.05) association as contributing factors for SM.

## 4. Discussion

The purpose of the present study was to assess the characteristics and practices of SM in Sargodha, Pakistan, during the COVID-19 pandemic and to shed some light on the potential factors contributing to the practices of SM. According to our results, around one-third of the selected population has practiced SM, meaning that the majority still preferred/tried to establish contact with a physician or a licensed healthcare professional before consuming any medicine. Hence, the drugs received and utilized via personal prescriptions were higher than the rate of SM. Our findings (34.3%) regarding the use of SM were similar to findings from other developing countries after the onset of the pandemic, such as studies conducted in Togo (34.2%) [32] and Nigeria [33,34]. The main sources of drugs for SM were leftover prescriptions procured from friends and family, receiving drugs directly from family, and requesting them directly OTC from a pharmacy; similar sources as easy access to medications and SM were documented in a recent study from Dhaka, Bangladesh [35], and from previous studies in Rio Grande, Brazil [36], and Kuwait [37].

In this study, SM practice showed that those working in medical fields might be more fearful about the adverse effects of taking drugs inappropriately [38]; the possible reason for this could be better accessibility to relevant and trustworthy COVID-related information (from their workplace or from the internet), both about the prevention and the treatment of the illness. These findings are in line with a study conducted in India [39], where greater drug-related knowledge has led to concerned attitudes towards SM. On the other hand, identical studies have also been published noting the opposite, i.e., with significant levels of comprehension of OTC and POM drugs, including their prescription and adverse reactions, healthcare professionals were more likely to self-medicate during the outbreak [40]. Among our respondents, over half had never had a COVID-19 test of any kind, while the majority of those who had tests were documented as negative. This finding could be due to having a good degree of self-awareness about their health among people with a higher educational status [41]. The reasons for SM reported in this study were the unavailability of physicians, fears or difficulties in getting in contact with them, or bad experiences/ineffective treatments associated with visiting them, which were noted in other reports as well [32,33,36,37]. Fears of contracting the virus and difficulties in travelling to healthcare facilities were similarly documented in a study conducted in Lahore, Pakistan [42], and Dhaka, Bangladesh [35].

Our study reports that azithromycin was the most commonly used POM during the COVID-19 pandemic, while other notable allopathic medicines were acetaminophen, being the most commonly used for SM, and cough syrups, which is consistent with other reports in the context of COVID and SM [42]. The reason for azithromycin SM could be due to its properties being effective against COVID in vitro in addition to its proposed property to alleviate inflammation of the respiratory epithelium [43]. Acetaminophen was also highly noted among participants as a preventive measure against COVID-19; this drug has a widespread use already in SM for various indications, however, its use has expanded remarkably during the viral outbreak, both for its classical and novel supposed indications [44,45]. Ivermectin was also used as a preventative measure during the pandemic, as some early reports suggested a more promising outcome associated with supplementing the drug [46]; nevertheless, no recent clinical study has been successful in reliably confirming the usefulness of this compound in the prevention of COVID-19 [47]. Hydroxychloroquine was also extensively used in the initial stages of the pandemic as a preventive measure against COVID-19; studies of a different nature and quality have described the productive use of hydroxychloroquine and azithromycin for hospitalized individuals [48]; however, the utilization of hydroxychloroquine alone or with azithromycin may lead to substantial cardiac toxicities—leading to lethal arrhythmias in hospitalized COVID-patients—and highlighting that the use of these drugs as SM is questionable at best [49]. When it comes to dietary supplements to prevent/treat COVID, Vitamin C was used by approximately one third of participants; some studies have noted the efficacy of Vitamin C in the management of COVID-19 [50]. Nevertheless, it is also important to note that in high doses and when taken for extended periods of time, this vitamin may cause unwanted and harmful effects, like kidney stones [51]. Similarly, there has been considerable interest in Vitamin D supplementation for the prevention of acute respiratory tract infections and, in turn, COVID-19 [52,53]; but being a lipid-soluble vitamin, one has to be mindful with dosage to prevent hypervitaminosis and its associated adverse outcomes. According to this study, the participants used herbal medicines, i.e., Senna Makhi Kehwa, for the treatment and anticipation of contracting COVID-19. This may be explained by the fact that traditional medicines are habitually utilized as a result of the accessibility and lower expenses associated with herbal products [54]. It is also worth mentioning that the WHO has invited development throughout the world, including medicines of natural origins and herbal products, to explore potential therapeutics for COVID-19 [55].

The practice of SM has been previously noted to be highly prevalent in association with several ailments, including for the treatment of chronic pain [56], toothache and other dental indications [57], gastro-intestinal issues [58], and mood disorders [59]; nonetheless, with the onset of the COVID-19 pandemic, an increase in the prevalence of SM drug use associated with respiratory tract infections was noted. The WHO has predicted that the COVID-19 pandemic may last for a number of years, resulting in serious socio-economic consequences and changes in individuals’ psycho-physical lifestyles, leading to deteriorating mental and physical health, which occurs in the backdrop of the unavailability of primary healthcare and mental care services [60]. With this in mind, national surveys on SM awareness and campaigns must be put forward to help educate laypeople and protect them from the potential harmful effects of the practice of SM.

The limitations of the present study must be acknowledged: firstly, the cross-sectional nature of the study design; the study was conducted in selected areas of Sargodha, with participants who were willing to participate in the research, which may have introduced bias into the results. Young adults and healthcare professionals are represented in high numbers among the participants. In this study, the practice of SM was associated with demographical patterns, i.e., age, gender, marital status, area of residence, and type of profession, however, this may not reflect the genuine image of SM in the entirety of Pakistan. Regarding statistical analyses, a limitation of the χ^2^-square test is its sensitivity to sample size. When a big enough sample is employed, even small associations may become statistically significant. When applying the χ^2^-square test, “statistically significant” does not automatically imply “meaningful”. To establish causality, a more thorough examination would be needed, which we aimed to amend with the introduction of univariate and multivariable logistic regression analyses. Finally, the main limitation in conducting the present research-based study was the limited time-frame available to complete the study.

## 5. Conclusions

Self-medication (SM) has become a significant issue of health and well-being in developing countries, which has been exacerbated by the presently occurring COVID-19 pandemic. This study has concluded that the practice of self-medication is undertaken by approximately one-third of the population in Sargodha. The major contributing factors towards SM during COVID-19 were the unavailability of physicians, the lack of effectiveness of medicines prescribed by the physicians, and the fear of contracting the virus. Based on our results, various allopathic and natural alternative medicines were used for the prevention and treatment of COVID-19: azithromycin, acetaminophen, Ivermectin, and vitamin C and D were the most frequently consumed medicines and supplements. Medical health professionals, having comprehensive knowledge about drugs, are mostly involved in practicing SM. To minimize SM, the public must consult with a physician before administering any type of drug to establish a reliable diagnosis and to get a prescription for POM with recommended dosages. One of the pertinent arms of intervention to minimize SM practice is to improve awareness against misinformation about illegal COVID-19 preventive products and aiming to improve psychological health in the pandemic crisis (thus reducing anxiety and the compulsion to perform SM). Consciousness and understanding about the possible adverse effects of SM must be established and validated on a continuous level; in addition, on a commercial level, collaboration from pharmacists not to sell products (especially POM) without a certified prescription must be developed and implemented.

## Figures and Tables

**Table 1:** Demographic characteristics and general information of participants.

Demographic Characteristics	Category	*n*, %
**Age (Years)**	18–28	339 (73.3)
	29–38	61 (13.5)
	39–48	32 (7.0)
	49–58	28 (6.2)
**Gender**	Male	270 (58.7)
	Female	190 (41.3)
**Marital Status**	Single	307 (66.7)
	Married	142 (30.9)
	Divorced	10 (2.4)
**Area of Residence**	Sargodha	166 (36.1)
	Peripheral part of the city	294 (63.9)
**Healthcare-Professional**	Yes	215 (46.7)
	No	245 (53.3)
**Tested for COVID-19**	Yes (the result was positive)	34 (7.2)
	Yes (the result was negative)	178 (38.7)
	No	248 (53.9)

**Table 2:** Characteristics of SM during the COVID-19 pandemic.

Descriptors			*p*-Values					
Variables	Categories	*n*, %	Age	Gender	Marital Status	Area of Residence	Medical Health Professional	Tested for COVID 19
**Did you practice SM?**	Yes	158 (34.3)	**0.042**	0.212	0.52	0.194	**0.046**	**<0.001**
	No	302 (65.7)						
**Types of medicines used**	Allopathic	71 (15.4)	N/A	0.892	N/A	**<0.001**	**0.05**	**<0.001**
	Herbal	104 (22.6)						
	Both	69 (15.0)						
	None	216 (47.0)						
**Did you use POM during SM?**	Yes	99 (21.5)	0.072	0.344	**0.004**	**0.002**	**0.001**	**<0.001**
	No	361 (78.5)						
**Sources of medicines**	From a prescription for a family member	48 (9.5)	N/A	0.798	0.255	0.147	0.294	**0.02**
	From a prescription for a friend	39 (7.7)	N/A	0.186	0.356	0.956	0.939	**<0.001**
	Requested directly from pharmacy	127 (25.0)	0.725	0.744	0.829	0.054	0.11	**0.004**
	Drugs from family members	53 (10.5)	N/A	0.248	0.219	0.593	**0.034**	0.235
	Drugs from friends	18 (3.5)	N/A	0.093	0.803	0.816	0.842	0.1
	Other	222 (43.8)	**0.186**	0.748	0.053	**0.001**	0.373	**<0.001**
**Were you aware of the possible side effects of the SM drugs?**	Yes	304 (65.9)	0.899	0.57	**0.005**	0.306	**<0.001**	**0.004**
	No	156 (34.1)						
**Did you receive information regarding the possible side effects of the SM drugs?**	No	104 (22.6)	N/A	**<0.001**	**0.049**	**<0.001**	**<0.001**	**0.001**
	Yes, from a physician	60 (13)						
	Yes, from a family member	100 (21.7)						
	Yes, from a colleague	23 (5.0)						
	Yes, from the internet	62 (13.5)						
	Yes, from other sources	111 (24.2)						
**Have you felt improvement in your symptoms due to above mentioned medicines/substances?**	Yes	284 (61.7)	**0.021**	0.456	**0.027**	0.738	**<0.001**	**0.036**
	No	176 (38.3)						
**Have you felt improvement in your symptoms due to above mentioned medicines/substances?**	Yes	259 (56.3)	**0.04**	**0.022**	0.41	**0.019**	**<0.001**	**0.006**
	No	201 (43.7)						
**Does pharmacist demand for a prescription when you visit to buy medicines?**	Yes	259 (56.3)	**0.04**	**0.022**	0.41	**0.019**	**<0.001**	**0.006**
	No	201 (43.7)						

*p* values ≤ 0.05 were presented in **boldface**; POM: prescription-only medicine.

**Table 3:** Medicines used as SM during the COVID-19 pandemic.

Descriptions			*p*-Values					
Variables	Categories	*n*, %	Age	Gender	Marital Status	Area of Residence	Healthcare Professional	Tested for COVID-19
Herbal medicines	Senna Makhi Kehwa	92 (20.0)	0.619	0.166	0.667	**<0.001**	**<0.001**	**0.017**
	Homeopathic medicine	79 (17.2)						
	None	238 (51.7)						
	Other	51 (11.1)						
Allopathic medicines	Azithromycin	110 (14.9)	0.468	**0.006**	0.195	0.371	**<0.001**	0.152
	Dexamethasone	54 (7.3)	N/A	0.331	0.429	0.856	0.347	**0.001**
	Hydroxychloroquine	81 (11.0)	0.004	0.108	0.645	0.059	**<0.001**	**<0.001**
	Ivermectin	13 (1.8)	0.375	0.833	0.186	0.055	**0.005**	**0.050**
	Acetaminophen	174 (23.6)	0.376	0.980	0.430	0.049	0.139	0.949
	Aspirin	45 (6.1)	**0.033**	0.652	**0.001**	0.065	0.212	0.587
	Stool softeners	42 (5.7)	0.312	0.153	0.387	0.354	0.156	0.652
	Cough syrups	96 (13)	0.600	0.5.37	0.177	**0.002**	**0.003**	0.102
	Unknown	121 (16.6)	0.905	0.387	**0.006**	0.813	**<0.001**	**<0.001**
Supplements	Vitamin D	47 (10.2)	0.190	0.451	0.307	0.347	**0.030**	0.318
	Vitamin C	180 (39.1)	0.772	**0.039**	0.618	**<0.001**	**0.004**	**<0.001**
	Folic acid	108 (23.5)	0.147	**0.032**	0.378	0.233	0.097	0.122
	Calcium	104 (22.6)	0.365	0.115	0.673	0.603	0.719	0.128
	Other	114 (24.8)	0.417	0.675	0.432	**0.005**	**<0.001**	**<0.001**

*p* values ≤ 0.05 were presented in **boldface.**

**Table 4:** Contributing factors and reasons associated with SM during the COVID-19 pandemic.

Variables	Categories	*n*, %	Age	Gender	Area of Residence	Healthcare Professional	Tested for COVID-19
**Reasons for SM**	Already existing habits	40 (7.3)	N/A	0.619	0.873	0.231	0.153
	Unavailability of the physician	76 (13.9)	0.096	0.506	**0.010**	0.713	0.710
	Financial issues	39 (7.1)	N/A	0.016	0.984	**0.006**	0.680
	Difficulty in travelling/reaching healthcare professionals	66 (12.0)	N/A	0.091	0.920	0.170	**0.030**
	Lack of effectiveness of medicines prescribed by physician	58 (10.6)	N/A	0.399	0.540	0.758	0.594
	Fear of contracting the virus	41 (7.5)	N/A	0.177	0.825	0.969	0.784
	Bad experience with physician	44 (8.0)	N/A	0.955	0.723	0.515	0.275
	Other	184 (33.6)	0.562	0.439	0.804	0.342	**0.002**

*p* values ≤ 0.05 were presented in **boldface**.

**Table 5:** Logistic regression analysis for identification of contributing factors for SM during COVID-19.

		Practice SM		Univariable Logistic Regression			Multivariable Logistic Regression		
		No	Yes	C.O.R	C.I	*p*-Values	A.O.R	C.I	*p*-Values
Variables	Categories	n (%)	n (%)						
**1. Age**	18–28	227 (67.0)	112 (33.0)	Ref		0.047 *	Ref	0.778–2.687	0.108
	29–38	33 (54.1)	28 (45.9)	1.72	0.990–2.987	0.054	1.446	0.146–1.051	0.244
	39–48	26 (81.3)	6 (18.8)	0.468	0.187–1.169	0.104	0.391	0.597–3.461	0.063
	49–58	16 (57.1)	12 (42.9)	1.52	0.695–3.322	0.294	1.438		0.418
**2. Healthcare professional**	Yes	131 (60.9)	84 (39.1)	1.482	1.007–2.181	0.046 *	1.163	0.740–1.828	0.513
	No	171 (69.8)	74 (30.2)	Ref			Ref		
**3. Tested for COVID-19**	Yes (the result was positive)	10 (29.4)	24 (70.6)	7.688	3.477–16.99	<0.001 *	5.258	2.261–12.229	<0.001 *
	Yes (the result was negative)	103 (57.9)	75 (42.1)	2.333	1.537–3.540	**<0.001**	1.884	1.189–2.987	<0.001 *
	No	189 (76.2)	59 (23.8)	Ref		**<0.001**	Ref		**0.007**
**4. Were you aware of the possible side effects of the SM drugs?**	**Yes**	181 (59.7)	122 (40.3)	2.266	1.463–3.508 *	<0.001 *	1.833	1.177–3.175	0.009 *
	**No**	121 (77.1)	36 (22.9)	Ref			Ref		
**5. Does pharmacist demand for a prescription when you visit to buy medicines?**	**Yes**	160 (61.8)	99 (38.2)	1.489	1.005–2.208 *	0.047 *	1.033	0.657–1.622	0.889
	**No**	142 (70.6)	59 (29.4)	Ref			Ref		
**6. Did you receive information regarding the possible side effects of the SM drugs?**	**No**	67 (64.4)	37 (35.6)	Ref		0.005 *	Ref		0.125
	**Yes, from a physician**	29 (48.3)	31 (51.7)	1.963	1.014–3.694	0.045 *	1.46	0.710–3.003	0.304
	**Yes, from a family member**	64 (64.0)	36 (36.0)	1.019	0.575–1.806	0.95	1.241	0.649–2.370	0.514
	**Yes, from a colleague**	15 (65.2)	8 (34.8)	1.068	0.556–2.056	0.844	1.235	0.583–2.641	0.581
	**Yes, from the Internet**	39 (62.9)	23 (37.1)	0.966	0.374–2.491	0.943	1.1	0.383–3.159	0.859
	**Yes, from other sources**	88 (79.3)	23 (20.7)	0.473	0.257–0.871	0.016 *	0.541	0.279–1.050	**0.069**
**Reasons**									
**7. Already existing habits**	**Yes**	26 (65.0)	14 (35.0)	1.032	0.523–2.038	0.928	-	-	-
	**No**	276 (65.7)	144 (34.3)	Ref			-	-	-
**8. Unavailability of the physician**	**Yes**	39 (51.3)	37 (48.7)	2.062	1.252–3.395	0.004 *	1.492	0.832–2.679	0.18
	**No**	263 (68.5)	121(31.5)	Ref			Ref		
**9. Financial issues**	**Yes**	27 (69.2)	12 (30.8)	0.837	0.412–1.701	0.623	-	-	-
	**No**	275 (65.3)	146 (34.7)	Ref			-	-	-
**10. Difficulty in travelling/reaching healthcare professionals**	**Yes**	35 (53.0)	31 (47.0)	1.862	1.099–3.156	0.021 *	1.086		0.793
	**No**	267 (67.8)	121 (32.2)	Ref			Ref	0.585–2.017	
**11. Lack of effectiveness of medicines prescribed by physician**	**Yes**	38 (65.5)	20 (34.5)	1.007	0.584–1.797	0.982	-	-	-
	**No**	264 (65.7)	138 (34.3)	Ref			-	-	-
**12. Fear of contracting the virus**	**Yes**	25 (61.0)	16 (39.0)	1.248	0.646–2.414	0.509	-	-	-
	**No**	277 (66.1)	142 (33.9)	Ref			-	-	-
**13. Bad experience with physician**	**Yes**	28 (63.6)	16 (36.4)	1.103	0.577–2.105	0.767	-	-	-
	**No**	274 (65.9)	142 (34.1)	Ref			-	-	-
**14. Other**	**Yes**	143 (77.7)	41 (22.3)	0.39	0.256–0.594	<0.001 *	0.639	0.380–1.074	**0.091**
	**No**	159 (57.6)	117 (42.4)	Ref			Ref		

C.O.R: Crude odds ratio (unadjusted OR), A.O.R: Adjusted OR, *: significant at ≤0.05, which are presented in **boldface**; multivariable logistic regression: adujstments were made for variables listed in the table for variables 1, 2, 3, 4, 5, 6, 8, 10 and 14.

## Data Availability

All data generated during the study are presented in this paper.

## Supplementary Materials

The following supporting information can be downloaded at: https://www.mdpi.com/article/10.3390/tropicalmed7110330/s1. Supplementary Material S1: Instrument for data collection.
