# Supplementary material for: Factors Associated with Self-Medication during the COVID-19 Pandemic: A Cross-Sectional Study in Pakistan
Source: Trop Med Infect Dis. 2022 Oct 25;7(11):330. doi: 10.3390/tropicalmed7110330 (PMC9694449; doi:10.3390/tropicalmed7110330)
Supplement: Supplementary file 1 [file tropicalmed-07-00330-s001.zip › tropicalmed-1890044-supplementary.pdf]

Self Medication Practices among Population Attending Community Pharmacies during COVID-19 Pandemic in Sargodha Pakistan

This research is being conducted in order to access the knowledge, attitude and practice to identify the contributing factors towards self-medication during COVID-19 pandemic. This questionnaire will require approximately 5 minutes to complete. All the information will be used for academic purpose only and accessible only by the research team. There is no any known risk posed to the participants and confidentiality will be maintained throughout the study. Your involvement is voluntary, and you are free to participate in or to withdraw from the study at any time.

SECTION A: Demographic and general information

1. Age __________Years
2. Gender ☐ Male ☐ Female
3. Marital Status ☐ Single ☐ Married ☐ Divorced ☐ Widowed ☐ Other
4. Area of Residence ☐ Sargodha ☐ Periphery
5. How do you classify your economic status?
☐ Lower class ☐ Lower middle class ☐ Upper middle class ☐ Upper class
6. Are you suffering from any underlying medical disorder?
☐ Hepatitis B ☐ Hepatitis C ☐ Diabetes mellitus ☐ Hypertension
☐ Any form of malignancy ☐ Chronic obstructive airways disease ☐ None
7. Are you a medical health professional? (doctor, medical student, nurse and other health professionalism) ☐ Yes ☐ No
8. Have you been tested for COVID-19?
☐ Yes (its positive) ☐ Yes (its negative) ☐ No

SECTION B:Assessment of Prevalence of Self-Medication During COVID-19 Pandemic

9. Did you medicate yourself in any form i.e. allopathic, herbal and supplements against Corona virus? ☐ Yes ☐ No
10. Have you taken any medication without a proper prescription written, as a precautionary measure in COVID-19 during last 03 months? (Allopathic/herbal medicines)
☐ Yes ☐ No

SECTION C:Identification of contributing factors towards Self-Medication During COVID-19 Pandemic

11. Does pharmacist demand for a prescription when you visit to buy medicines?
☐ Yes ☐ No
12. Are you aware that such medicines\ substances can affect your health adversely?
☐ Yes ☐ No
13. What was/were reason to take medicine without doctor's prescription? (mark all that apply)
☐ It is a habit of yours ☐ Unavailability of doctor as required ☐ Financial Issue
☐ Difficulty in travelling to doctor ☐ Lack of effectiveness of doctor's medicine
☐ Fear of getting contact to virus ☐ Bad experience with doctor ☐ Other
14. Who/What influenced you to take medicine without meeting a doctor?(mark all that apply)
☐ Yourself ☐ Doctor ☐ Family member ☐ Internet/Social media ☐ Colleague ☐ Other

SECTION D:Assessment of Knowledge, Attitudes and Practices of Self-Medication During COVID-19 Pandemic

15. What was/were the type/s of medicine have you taken as a preventive measure?
☐ Allopathic ☐ Herbal ☐ Both ☐ None

16. What was/were the symptoms you expected to be prevented?(mark all that apply)
☐ Fever ☐ Cough ☐ Flu ☐ Abdominal burn/pain ☐ Shortness of breath
☐ Headache ☐ Loss of sense of taste/smell ☐ Sore throat ☐ Diarrhoea
☐ Anxiousness ☐ Constipation ☐ Body pain ☐ Allergies ☐ None

17. How did you get the medicine/substances as a precautionary measure?(mark all that apply)
☐ Prescription written for a family member ☐ Prescription written for a friend
☐ Directly requested from the pharmacy ☐ Drugs bought for a family member
☐ Drugs bought for a friend ☐ Other

18. What is/are the medicines you took as a precautionary measure in COVID-19?(mark all that apply)
☐ Azithromycin ☐ Dexamethasone ☐ Hydroxychloroquinone ☐ Ivermectin
☐ Panadol ☐ Disprin ☐ Soften (anti-allergic) ☐ Cough syrup ☐ Unknown

19. Was/Were your symptoms improved with medicine/substances?
☐ Yes ☐ No

20. How often do you take medicines/substances in below mentioned way?
☐ Once a week ☐ Once in two weeks ☐ Once a month ☐ Other

21. Have you felt improvement in your health due to above mentioned medicines/substances?
☐ Yes ☐ No

22. What are the adverse effects faced you by using the medicines?(mark all that apply)
☐ Diarrhoea ☐ Chest discomfort ☐ Skin rash ☐ joint and muscle pain ☐ Chills
☐ Vomiting/nausea ☐ Dry mouth ☐ Nasopharyngitis ☐ Other

23. What is/are the herbal medicines you took in COVID-19?(mark all that apply)
☐ Senna Makhi Kehwa ☐ Homeopathic medicines ☐ None ☐ Other

24. How many times have you used them?(mark all that apply)
☐Daily ☐ Once a week ☐ Twice a week ☐ Once a month ☐ On & Off ☐Continuously

25. Do you believe that above herbal medicine can boost your immunity?
☐ Yes ☐ No ☐ Not sure

26. Have you taken any supplements mentioned below in COVID-19? (Mark all that apply)
☐ Vitamin D ☐ Vitamin C ☐ Surbex Z ☐ Calcium ☐ Other

27. How many times have you been using the supplements?
☐ Daily ☐ Once a week ☐ Twice a week ☐ Once in a month
☐ On and Off ☐ Continuously ☐ Other

28. Do you believe that the supplements can boost your immunity?
☐ Yes ☐ No ☐ Not sure

Thank you
